# Influence of Various Desensitizing Mouthrinses and Simulated Toothbrushing on Surface Roughness and Microhardness of Tetric N-Ceram Bulk-Fill Resin Composite: An *In Vitro* Study and Scanning Electron Microscope Analysis

**DOI:** 10.1055/s-0041-1739547

**Published:** 2022-02-17

**Authors:** Paras Mull Gehlot, Parvathi Sudeep, Vinutha Manjunath, B.M. Annapoorna, L. Krishna Prasada, Bhojraj Nandlal

**Affiliations:** 1 Department of Conservative Dentistry and Endodontics, JSS Dental College and Hospital, JSS Academy of Higher Education and Research, SS Nagara, Mysuru, Karnataka, India; 2 Department of Conservative Dentistry and Endodontics, KVG Dental College and Hospital, Dakshina Kannada, Karnataka, India; 3 Department of Pedodontics and Preventive Dentistry, JSS Dental College and Hospital, JSS Academy of Higher Education and Research, SS Nagara, Mysuru, Karnataka, India

**Keywords:** bulk-fill, direct composite resins, mouthrinses, desensitizer, surface roughness, microhardness

## Abstract

**Objectives** Bulk-filled composite resins are popularly used for posterior restorations due to various advantages. Routine oral hygiene measures like toothbrushing and the use of various mouthrinses can influence the mechanical properties of composite resins. Desensitizing mouthrinses are widely used as well, to manage dentinal hypersensitivity. Studies on the influence of desensitizing mouthrinses on bulk-filled composites are limited. Hence, the objective of the present *in vitro* study was to evaluate the influence of toothbrushing and various desensitizing mouthrinses on the surface roughness and microhardness of Tetric N-Ceram bulk-fill composite resin.

**Materials and Methods** Fifty Tetric N-Ceram bulk-fill composite resin disks were prepared and were randomly divided into five groups ( *n* = 10). Group 1 (Control): no toothbrushing and no mouthrinse; Group 2: toothbrushing only; Group 3: toothbrushing + HiOra-K mouthrinse; Group 4: toothbrushing + Listerine Sensitive mouthrinse; and Group 5: toothbrushing + Shy-OR mouthrinse. The specimens were brushed with a soft bristle brush using a toothpaste slurry and immersed in respective mouthrinse twice daily for 1 month. The mean surface roughness (average roughness) and microhardness (Vickers Pyramid number) values were determined and the data were tabulated. Data were analyzed using one-way analysis of variance, Post-hoc Tukey test, and Pearson correlation test. A *p* -value less than 0.05 was considered statistically significant.

**Results** Specimens treated with HiOra-K mouthrinse exhibited maximum surface roughness ( *p* < 0.05) and specimens treated with Listerine Sensitive exhibited the least microhardness ( *p* < 0.05). A weak negative correlation was found between surface roughness and microhardness for groups 1, 2, and 5, while a weak positive correlation was found for groups 3 and 4.

**Conclusions** It is suggested that desensitizing mouthrinses containing alcohol or essential oils can lead to increased surface roughness and reduction in microhardness of bulk-fill composites, which could have an undesirable effect on their clinical performance.

## Introduction

Direct composite resins are popular aesthetic restorative materials of choice in anterior teeth. 1 However, increasing aesthetic demands and constant evolution in material science has led to the development of posterior composites. 2

Over the past few years, “bulk-fill” composites have become popular as a posterior restorative material, which enables bulk placement and curing of up to 4 mm thickness in a single step. 3 The manufacturers claim that this technique is less time consuming and it also reduces the polymerization shrinkage. 4 5 Tetric N-Ceram bulk-fill (Ivoclar Vivadent AG, Schaan/Liechtenstein) is one such high-viscosity universal bulk restorative composite resin with high filler content. The manufacturer states that it has a shrinkage stress reliever to lower polymerization shrinkage. 5

Since composite resins are polymer based, they could easily degrade when exposed to oral conditions. 6 Food and beverages may degrade the surface of the resins and alter their surface hardness by affecting the organic component of the resin matrix. Furthermore, oral hygiene practices like toothbrushing and the use of various mouthrinses could have an impact on the mechanical and surface properties of the composite resins. 2 7

Various mouthrinses, advocated for chemical plaque control, have been known to negatively influence the properties of resin composites. 6 8 Among the various chemical components present in the mouthrinses, alcohol has been reported to be responsible for the degradation of the resin component. 6 9 Desensitizing mouthrinses have also been prescribed in an attempt to reduce dentinal hypersensitivity. They have demonstrated a significant reduction in sensitivity. 10

Various studies have evaluated the effect of mouthrinses and beverages on the surface degradation of various composite resins and bulk-fill resin composites. 11 12 13 However, to the knowledge of the authors, there is no study on the influence of toothbrushing and desensitizing mouthrinses on the surface roughness and microhardness of Tetric N-Ceram bulk-fill composite. Surface roughness and hardness could influence the survival of composite restorations as well as the decision of clinicians for a replacement. 12

The aim of the present *in vitro* study is to evaluate the influence of three commercially available desensitizing mouthrinses and simulated toothbrushing on surface roughness and microhardness of Tetric N-Ceram bulk-fill resin composite. The null hypothesis tested is that there is no influence of desensitizing mouthrinses on the surface roughness and microhardness of Tetric N-Ceram bulk-fill composites.

## Materials and Methods

This *in vitro* study was approved by the Institutional Ethical Committee for research on human subjects or specimens. The materials used in the study and their compositions are presented in Table 1 . pH of the three mouthrinses was determined using a digital pH meter (Mettler-Toledo India Ltd., Mumbai, India).

**Table 1:** Materials used in the present study

Materials	Composition
Tetric N-Ceram Bulk-Fill (IVA) (Ivoclar Vivadent AG, Schaan/Liechtenstein) X48457	• Matrix: Bis-GMA, Bis-EMA, and UDMA; standard filler Ba-Al-Si glass with 2 mean filler size • Isofiller: Ytterbium fluoride, mixed oxides, additives, catalyst, stabilizers, and pigments • Loading: 75–77% by weight (53–55% by volume)
HiOra-K Mouthwash For Sensitive Teeth (The Himalaya Drug Company, Hyderabad, India) Ayurvedic Proprietary Medicine	• Powders: Suryakshara (potassium nitrate), peppermint satva ( *Mentha piperita* ) • Oils: Tailaparnah ( *Eucalyptus globulus* ), Tvak ( *Cinnamomum zeylanicum* ), Jatiphala ( *Myristica fragrans* ), Misreya ( *Foeniculum vulgare* ), Barbari ( *Ocimum basilicum* ), Lavanga ( *Syzygium aromaticum* /Clove). • Others: Sodium benzoate, bronopol, potassium sorbate, saccharin sodium (pH = 4.60)
Listerine Sensitive (Johnson & Johnson Limited, Maidenhead, United Kingdom) N-564110	• Aqua, sorbitol, alcohol, potassium nitrate, poloxamer 407, benzoic acid, sodium saccharin, eucalyptol, aroma, methyl salicylate, thymol, sucralose, sodium benzoate, menthol, sodium fluoride, Cl 42053, sodium fluoride (0.022% w/v 100 ppm fluoride) • pH = 4.54
Shy-OR (Group Pharmaceuticals, Malur, India) 8902958001851	• Potassium nitrate 3%, triclosan 0.3%, and sodium fluoride 0.2% • Other: Xylitol • pH = 5.4

Abbreviations: Bis-EMA, bisphenol ethyl methacrylate; Bis-GMA, bisphenol glycol dimethacrylate; UDMA urethane dimethacrylate.

### Specimen Preparation

The sample size for the present study was determined based on previous studies 6 12 14 using a power analysis program (G* Power, Heinrich Heine University, Düsseldorf, Germany), which was determined to be 50 with a 0.5% confidence interval. Fifty cylindrical specimens (8 mm in diameter and 2 mm in height) of Tetric N-Ceram bulk-fill composite resin were prepared using a Teflon mold. The composite was manipulated according to the manufacturer's instructions. A transparent matrix strip was placed over the composite resin and gently pressed with a glass slide to obtain a flat and void-free surface. The top surface was cured using a light-emitting diode curing device (Bluephase C8, Ivoclar Vivadent AG, Schaan/Liechtenstein) at 1,200 mW/cm ^2^ power density, for 40 s.

Once cured, the resin specimens were stored in distilled water for 24 h at 37°C in a dark environment. The specimens were subsequentially polished (20 s for each step) using Sof-Lex™ spiral finishing and polishing wheels (3M ESPE, St. Paul, Minnesota, United States) with a mild uniform intermittent pressure and slow-speed handpiece with water cooling. All finishing and polishing procedures were accomplished at the low speed of 10,000 rpm by one investigator.

The 50 specimens were randomly divided into five groups ( *n* = 10). Group 1 (Control): no toothbrushing and no mouthrinse used, specimens stored in distilled water; Group 2: specimens with toothbrushing only and immersion in distilled water; Group 3: specimens with toothbrushing and immersion in HiOra-K mouthrinse (The Himalaya Drug Company. Hyderabad, India); Group 4: specimens with toothbrushing and immersion in Listerine Sensitive mouthrinse (Johnson & Johnson Limited, Maidenhead, United Kingdom); and Group 5: specimens with toothbrushing and immersion in Shy-OR mouthrinse (Group Pharmaceuticals, Malur, India).

### Toothbrushing and Mouthrinsing Protocol

The specimens were manually brushed with a fluoride-containing toothpaste slurry (Colgate-Palmolive [India] Limited, Mumbai) every day for 2 min twice a day for 30 days. 15

For the slurry, 200 mg of fluoridated toothpaste (1,000 ppm fluoride) was weighed on a digital weighing machine (Shimadzu Corporation, Japan) and mixed with water in a ratio of 1:3 by weight and stirred in a container. 15 16 Using a soft bristle toothbrush (Oral-B Sensitive, Gillette India Limited, P&G Plaza, Mumbai, India), the toothpaste slurry was applied to the composite specimens. The bristles were vertically oriented and the specimen surfaces were brushed in a horizontal back and forth motion (100 strokes/min) by a single operator. 17 The surfaces of all specimens were aligned in the same plane to ensure uniform abrasion while toothbrushing; the toothbrushes were replaced every week. At the end of brushing, the specimens were washed under running water to remove the toothpaste.

Subsequently, the specimens were immersed in 10 mL of respective desensitizing mouthrinse and agitated at room temperature for 2 min twice a day for 30 days. 16 18 19 The specimens were stored in distilled water at 37°C between the cycles and at completion of each cycle.

### Surface Roughness Analysis

**Fig. 1:**
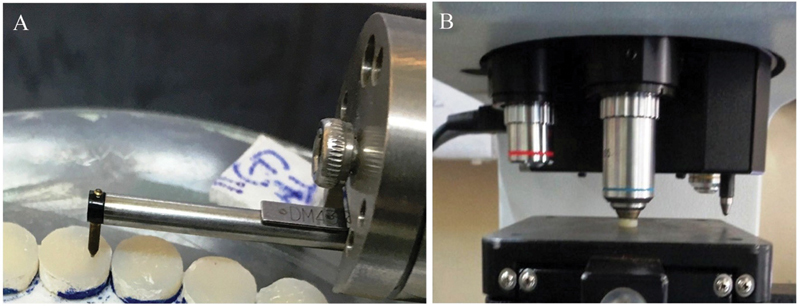
( **A** ) Surface roughness evaluation: Digital Surface roughness tester (Surfcom Flex, Carl Zeiss Industrial Metrology, GmbH. Germany. ( **B** ) Vickers microhardness test: Vickers hardness testing machine (HWMMT–XT; Highwood).

The surface roughness measurements were taken using a digital surface roughness tester (Surfcom Flex, Carl Zeiss Industrial Metrology, GmbH, Germany) fitted with a diamond stylus (tip radius: 2 µm, measuring force of 0.75 mN with 4 mm traversing length, at the drive speed of 1 mm/s). The average roughness (Ra, µm) was determined on the top surface in the middle of the specimen close to the centerline and perpendicular to the toothbrushing direction ( Fig. 1A ). The Ra of three readings were calculated for each specimen and data tabulated.

### Microhardness Testing

The Vickers hardness (VH) was tested with a diamond micro-indenter (Vickers hardness testing machine, HWMMT–XT; Highwood) at the top surface of each specimen and a load of 100 g was applied with a 15 s dwell time at room temperature 20 ( Fig. 1B ). The VH for each specimen surface was recorded as the average of three random indentations and the values were tabulated.

The formula used for determining the VH (kgp/mm ^2^ ) is:



where, *P* is the load applied in kilogram force (kgf) and *d* the average length in mm of the diagonals.

### Scanning Electron Microscope (SEM) Analysis

One specimen from each group was randomly selected for observation of the morphology of composite degradation using a scanning electron microscope (SEM [EVO LS15, Carl Zeiss Microscopy, GmbH, Gottingen, Germany]) with accelerated voltage of 15 kV. The SEM images were captured at the magnification of 1,500–2,000 × .

### Statistical Analysis

The mean surface roughness and microhardness (kgp/mm ^2^ ) of the composite specimens after toothbrushing and mouthrinsing protocol were estimated and statistically analyzed with one-way analysis of variance (ANOVA) and Tukey's post-hoc test. Pearson correlation was used to determine the relationship between surface roughness and microhardness of respective groups. The *p* -value of 0.05 was considered statistically significant. All statistical analyses were performed with the Statistical Package for the Social Sciences (SPSS 23.0, IBM Corp, United States).

## Results

### Surface Roughness

**Graph 1:**
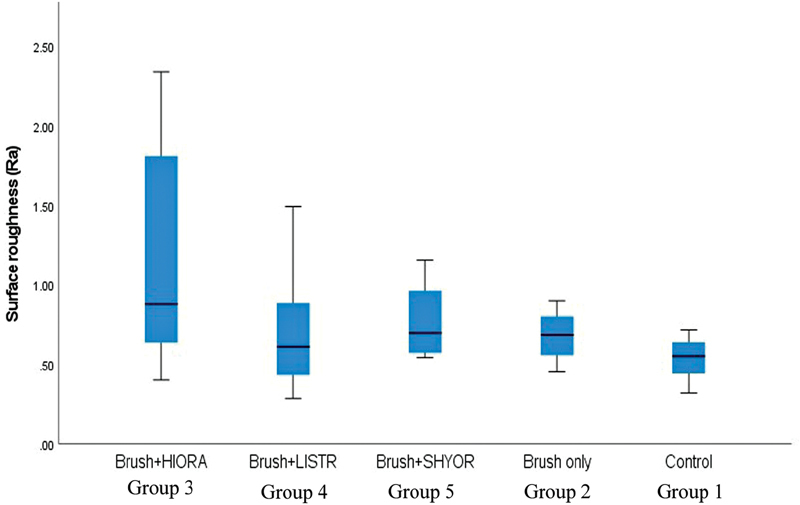
Box-plot graph comparing mean surface roughness among different groups.

The mean surface roughness (standard deviation) of composite specimens after various treatments are shown in Table 2 . The surface roughness, in the ascending order, was Group 1 < Group 2 < Group 4 < Group 5 < Group 3. The results of ANOVA revealed statistically significant differences among the groups ( *p* = 0.014). The Tukey post-hoc test revealed a statistically significant difference between group 3 (HiOra-K) and other groups. Graph 1 represents the box plots comparing the surface roughness of various groups.

**Table 2:** Mean (standard deviation) of surface roughness and microhardness

Groups ( *n* = 10)	Surface roughness (Ra values) μm	Microhardness HV (Kgp/mm ^2^ )
Group 1 (Control)	0.5305 (0.11947) ^B^	91.80 (11.305) ^A^
Group 2 (Toothbrushing only)	0.6747 (0.14067) ^B^	75.80 (3.120) ^B^
Group 3 (Toothbrushing + HiOra-K)	1.1324 (0.66931) ^A^	70.30 ^Aa^ (2.111) ^C^
Group 4 (Toothbrushing + Listerine Sensitive)	0.7382 (0.41999) ^B^	61.80 (3.120) ^D^
Group 5 (Toothbrushing + Shy-OR)	0.7614 (0.21763) ^B^	63.20 (1.989) ^D^
ANOVA	*F* = 3.52 *p* = 0.014	*F* = 178.89 *p* = 0.000

Abbreviations: ANOVA, analysis of variance; HSD, honest significant difference; HV, Vickers Pyramid number; Ra, average roughness.

Note: Capital letter superscripts indicate comparison within different groups for surface roughness and microhardness (One-way ANOVA and Tukey HSD post-hoc; Significance: *p* < 0.05).

### Microhardness

**Graph 2:**
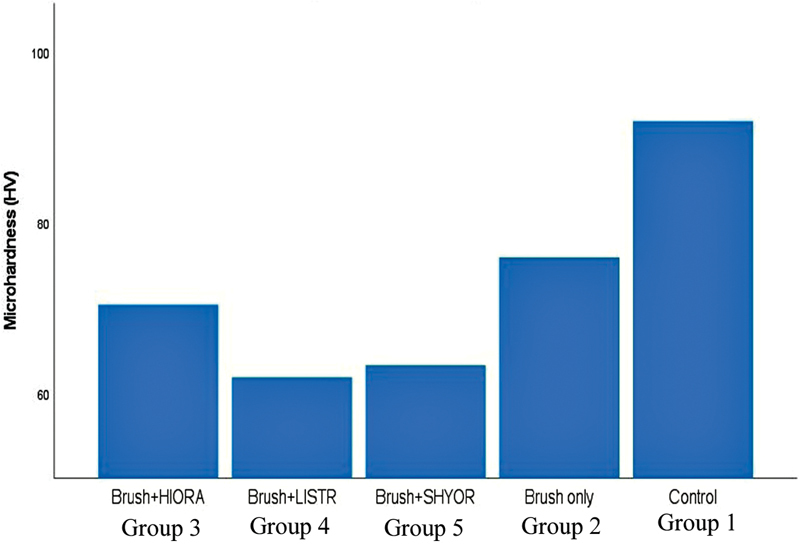
Bar graph comparing mean microhardness (Kgp/mm ^2^ ) among different groups.

The mean microhardness (standard deviation) of various groups is tabulated in Table 2 . Statistically significant differences were observed in the microhardness values among the groups ( *p* = 0.000). The microhardness values in ascending order were Group 4 < Group 5 < Group 3 < Group 2 < Group 1. The Tukey post-hoc test revealed a statistically significant difference between groups 1, 2, and 3; groups 4 and 5 were statistically similar. Graph 2 represents the comparison among the microhardness values of various groups.

The Pearson correlation between the surface roughness and microhardness revealed a weak negative correlation for groups 1 ( *r* = –0.807), 2 ( *r* = –0.284), and 5 ( *r* = –0.664). However, a weak positive correlation was found for groups 4 ( *r* = 0.151) and 3 ( *r* = 0.226).

### SEM Evaluation

**Fig. 2:**
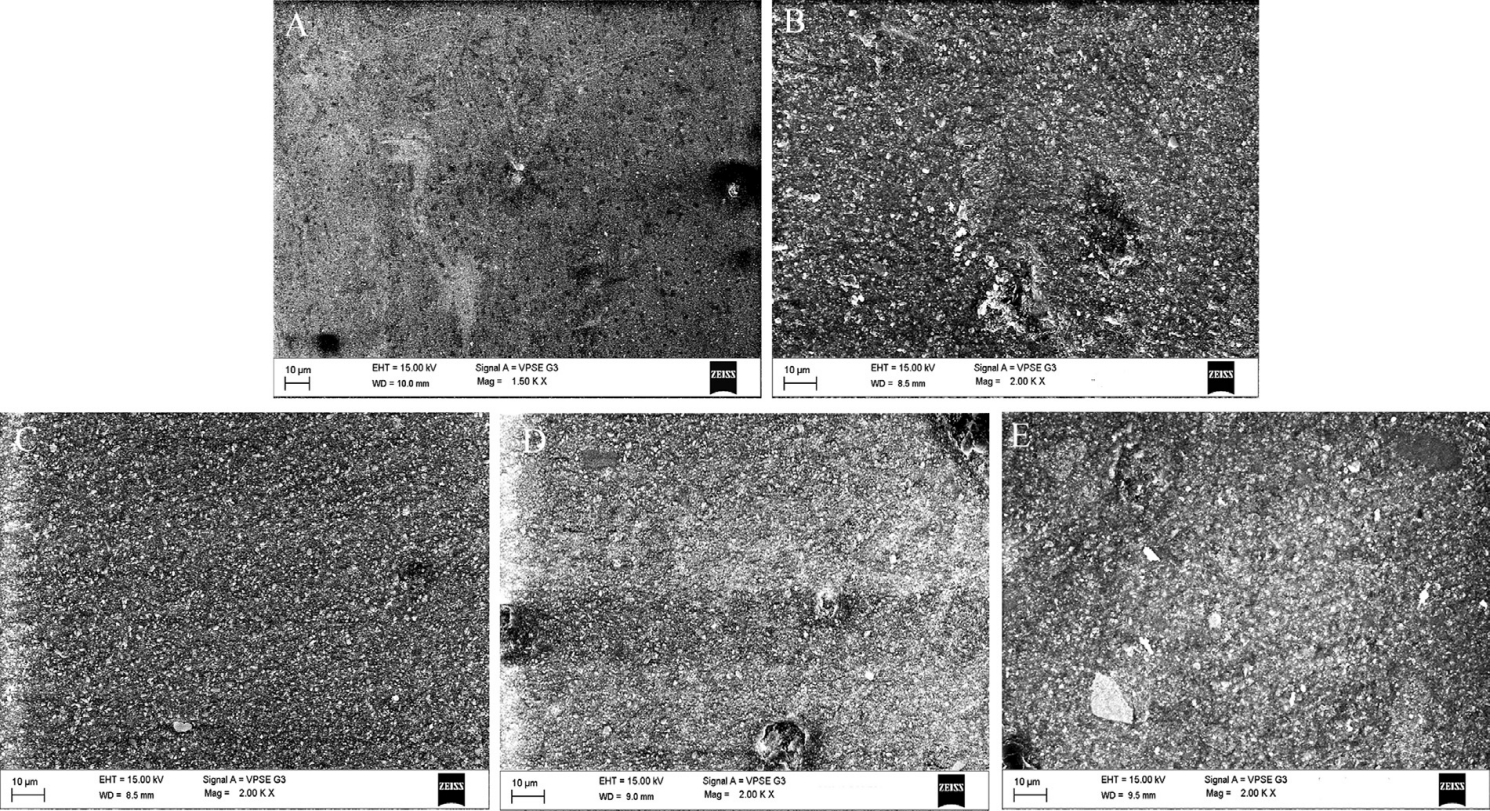
Tetric N-Ceram Bulk-Fill SEM photomicrographs at 1,500–2,000× magnification. ( **A** ) Group 1 (control), ( **B** ) Group 2 (Toothbrushing only), ( **C** ) Group 3 (Toothbrushing + HiOra-K), ( **D** ) Group 4 (Toothbrushing + Listerine Sensitive), and ( **E** ) Group 5 (Toothbrushing + Shy-OR).

The changes in the superficial topography due to toothbrushing and mouthrinse usage and their association were qualitatively evaluated under the SEM at 1,500–2,000× magnification ( Fig. 2 ). Group 1 (control) demonstrated a smooth and intact surface and the organic matrix was undisturbed. However, areas of voids and the Sof-Lex spiral detachment particles were visible ( Fig. 2A ). In group 2 (toothbrushing only), the abrasive effect of toothbrushing had caused pitting due to dislodgement of filler (small) particles. The free filler particles were visible. However, no dissolution of the organic matrix was noted ( Fig. 2B ). Group 3 (toothbrushing + HiOra-K) demonstrated significant pitting due to dislodgement of filler along with some areas of matrix dissolution ( Fig. 2C ). Group 4 (toothbrushing + Listerine Sensitive) demonstrated larger filler particle debonding and some patches of organic matrix dissolution ( Fig. 2D ). Group 5 (toothbrushing + Shy-OR) surface also demonstrated moderate filler dislodgement and multiple shallow patches of the organic matrix dissolution ( Fig. 2E ).

## Discussion

Patients experience tooth sensitivity due to loss of enamel or cementum, or due to dental bleaching procedures. Mouthrinses are frequently prescribed by dentists as a minimally invasive approach for the management of dental hypersensitivity. The objective is to seal the exposed dentinal tubules (like fluoride application) or desensitize the nerve endings with sedative agents (like potassium nitrate). However, many over-the-counter mouthrinses are used by patients without medical supervision. Along with their beneficial effects, they could also have side effects on dental tissues and could affect the longevity of the restoration. 21 22

This study attempted to evaluate the short-term effects of using desensitizing mouthrinses along with simulated toothbrushing on the surface of a bulk-filled composite. Based on the results of the present study, the null hypothesis was rejected since there were statistically significant differences between the control and the test groups, although this was not applicable for all the test groups.

Surface roughness evaluation is of clinical importance. The surface texture and polishing protocol of restorative materials have an influence on plaque adherence, wear properties, aesthetics, and surface discoloration of composite resins. 23 It has also been reported that bulk-fill composites are rougher than nano-filled composites. 23 24 25 Hence it is important to finish and polish all resin composite restoration with a suitable system. In the present study, all specimens were finished and polished with Sof-Lex spiral wheels, which are an accepted system for bulk-fill composites. 23 24 Roughness is related to irregularities and it is usually evaluated as Ra, which is defined as the mean arithmetical value of all absolute distances of the profile inside of the measuring length. 26 The objective of surface finishing and polishing after composite restoration is to match the roughness obtained after enamel-to-enamel contact in occlusal areas, which is ∼0.64 µm. 27

The surface roughness data for group 1 was acceptable; however, for group 2 it was slightly more than acceptable, and for the test groups it was not acceptable ( Table 2 ). This difference in the surface roughness between groups could be attributed to various reasons.

Toothbrushing action causes abrasion of the polymer matrix, leading to the surface roughness of the composite. This is mainly attributed to filler exposure and loosening of filler particles. 2 7 The composition of composite resin has an important effect on the surface roughness. 23 24 To avoid this bias, in the present study a single composite was studied. However, according to Martos et al, the mechanism of hydrolytic degradation in the presence of solvents is enhanced, especially in the presence of filler particles with metallic ions like barium and zinc. The reason for this is the electropositive nature of these ions and their ability to react with the aqueous solution. 28 Tetric N-Ceram also contains barium in the fillers among other ions and this could have contributed to degradation. The sorption and solubility of composite resins when in contact with mouthrinses have also been studied as a cause for degradation. 29 The probable elution of the unreacted monomers and a degrading effect on the polymer chain, after exposure of composite to chemicals, water, artificial saliva, alcohol, solvents, acids, or alkali, lead to increased plasticization. 16 18 The high sorption of triethylene glycol dimethacrylate and bisphenol glycol dimethacrylate (Bis-GMA) is due to the ether linkages and hydroxyl groups, respectively. The composition of Tetric N-Ceram bulk-fill monomer consists primarily of Bis-GMA resin, which although hydrophobic is still susceptible to the chemical reaction by alcohol. 29

In the present study, the specimens immersed in HiOra-K had the highest surface roughness. This is attributed to the presence of eugenol and other herbal oils in the mouthrinse that may have softened the polymer matrix. 30 It is reported that alcohol-containing mouthrinses showed higher sorption and solubility since they penetrate the polymer network, causing expansion of the polymer structure. This allows the release of residual monomers, causing dissolution of the linear polymer chain, leading to subsurface and surface degradation of the composites. 29 31 However, in the present study, the surface roughness in the group with Listerine Sensitive (alcohol containing) was not statistically different compared with control. The quantitative surface roughness data corresponded with the qualitative evaluation using SEM ( Fig. 2 ).

The pH of the solvent (mouthrinse) affects the sorption and solubility behavior of the composite resin, which can influence the hydrophilicity of the matrix and the chemical composition of the filler. A lower pH may have a greater softening effect on the resin matrix (Bis-GMA) or hydrolysis of the silane coupling agent, and could promote the dislodgement of filler particles, causing increase in surface roughness. 13 32 The lower pH of HiOra-K and the essential oil contents could have caused the degradation of resin matrix of Tetric N-Ceram bulk-fill (Bis-GMA) leading to accelerated surface roughness and reduction in surface microhardness. 8 13 14

A study by Lopes et al found that the mean surface roughness for Tetric N-Ceram bulk-fill following toothbrushing was 0.49 and 0.69 at 1 year and 2 years of brushing duration, respectively. However, in the present study, the roughness values of 0.67 were obtained for a 1-month toothbrushing cycle, which could be attributed to the difference in the study design and materials. 1 Similarly, Yilmaz and Mujdeci found that the surface roughness values of nanohybrid composites when exposed to mouthrinses containing alcohol and essential oils were 0.1 to 0.092 and 0.003 to 0.011, respectively. The study also found that mouthrinses containing both alcohol and essential oils had the maximum surface roughness values (0.17–0.2). 14 However, in the present study, the Ra of bulk-fill composite was 0.74 and 1.13 when using mouthrinses containing alcohol and essential oils, respectively. This difference can be attributed to the type of composite and the additional toothbrushing protocol used in the present study.

Hardness is a mechanical property related to a material's resistance to wear, and is usually measured using Vickers or Knoop hardness method. 2 20 These methods are popular since they are simple, nondestructible, and repeatable. 20

The effect of mouthrinses on hardness and wear is material dependent (composition and filler type). 30 Most of the studies comparing the hardness values among various composites have attributed the reduction in hardness due to less filler content. 20 However, reduction in the hardness among the groups of the present study could be attributed to the chemical degradation of the composite surfaces related to the resin matrix as a single composite was studied. 33 The immersion in desensitizing mouthrinse may have, along with toothbrushing, altered the resin matrix, causing exposure of the filler particles, leading to alteration in the hardness of the resin surfaces. 1 The type of chemical and the duration of exposure are important determinants that may affect the hardness of the composite. In the present study, the microhardness values were statistically different except between groups 4 and 5. The reduction in hardness value found in Listerine Sensitive and HiOra-K could be attributed to the presence of alcohol and phenolic compound, respectively, which cause greater sorption and solubility of the composite. 9 11 16 34

According to Tanthanuch et al, the baseline microhardness values of Tetric N-Ceram in contact with various food-stimulating agents and beverages varied from 71.27 to 75.95 kgp/mm ^2^ ; similar values were obtained for the toothbrushing group in the present study too. 13 However, the microhardness for specimens in contact with mouthrinses ranged from 61.80 to 70.30 kgp/mm ^2^ . This difference could be attributed to various factors like the type of solution or the restorative material tested, the duration of immersion, and toothbrushing protocol.

Other reasons that cause reduction in the surface microhardness include low pH of the mouthrinses, which soften the matrix and cause surface degradation due to various reasons mentioned previously. 11 13 16 30 35

The reduction in the hardness of specimen immersed in Shy-OR mouthrinse could not be established. According to Gürgan et al, both alcohol-free and alcohol-containing mouthrinse can reduce the microhardness of restorative materials. 34 The role of xylitol (sugar substitute) on the mechanical properties of composite resins needs to be evaluated since this was one of the components in Shy-OR.

The correlation of surface roughness and microhardness was analyzed, which revealed different values for different groups. A negative correlation was found for group 1 (control), group 2 (toothbrushing), and group 5 (toothbrushing + Shy-OR), which is in agreement with a previous study. 32 However, a positive correlation was found in group 3 (HiOra-K) and group 4 (Listerine Sensitive), in which both mouthrinses contained phenol or alcohol. This difference in the correlation among the groups can be associated with the fluoride content of the toothpaste or the chemical components of the mouthrinse. Different solutions can alter the ratio of organic and inorganic content of the composites since the resin matrix and filler particles do not abrade to the same degree. 8 36 37

The limitations of the present study include (i) the *in vitro* nature of the study, (ii) comparatively a small sample size, (iii) the composite disk along with manual toothbrushing cannot completely simulate the oral environment, and (iii) the nonavailability of toothbrushing simulation machine. Hence, caution must be taken when extrapolating the results to clinical situations. More *in vivo* studies on different bulk-filled composites and different mouthrinses are needed to confirm the results of the present study.

## Conclusions

Within the limitations of the present study, it can be suggested that desensitizing mouthrinses like HiOra-K and Listerine Sensitive have a deteriorating effect on the surface roughness and microhardness of Tetric N-Ceram bulk-fill composite. Hence, it may be advisable for patients with dentinal hypersensitivity and extensive bulk-fill composite restorations to avoid desensitizing mouthrinses containing alcohol or essential oils.
